# Local treatment of cancellous bone grafts with BMP-7 and zoledronate increases both the bone formation rate and bone density: A bone chamber study in rats

**DOI:** 10.3109/17453674.2011.566138

**Published:** 2011-04-05

**Authors:** Ola Belfrage, Gunnar Flivik, Martin Sundberg, Uldis Kesteris, Magnus Tägil

**Affiliations:** Department of Orthopedics, Clinical Sciences Lund, Lund University, Sweden

## Abstract

**Background and purpose:**

The remodeling of morselized bone grafts in revision surgery can be enhanced by an anabolic substance such as a bone morphogenetic protein (BMP). On the other hand, BMPs boost catabolism and might cause a premature resorption, both of the graft and of the new-formed bone. Bisphosphonates inactivate osteoclasts and can be used to control the resorption. We studied a combination of both drugs as a local admix to a cancellous allograft.

**Methods:**

Cancellous bone allografts were harvested and freeze-dried. Either saline, BMP-7, the bisphosphonate zoledronate, or a combination of BMP-7 and zoledronate were added in solution. The grafts were placed in bone conduction chambers and implanted in the proximal tibia of 34 rats. The grafts were harvested after 6 weeks and evaluated by histomorphometry.

**Results:**

Bone volume/total volume (BV/TV) was 50% in the grafts treated with the combination of BMP-7 and zoledronate and 16% in the saline controls (p < 0.001). In the zoledronate group BV/TV was 56%, and in the BMP group it was 14%. The ingrowth distance of new bone into the graft was 3.5 mm for the combination of BMP-7 and zoledronate and 2.6 mm in the saline control (p = 0.002). The net amount of retained remodeled bone was more than 4 times higher when BMP-7 and zoledronate were combined than in the controls.

**Interpretation:**

An anabolic drug like BMP-7 can be combined with an anti-catabolic bisphosphonate as local bone graft adjunct, and the combination increases the amount of remaining bone after remodeling is complete.

The clinical results of revision hip arthroplasties using morselized and impacted allografts are good (Ornstein et al. 2009). There are indications from radiostereometric studies that the graft does not always provide full stability during remodeling, and a continuous migration of the cup has been shown after as long as 5 years in 5/17 patients (Ornstein et al. 2006). Morselized and impacted allograft is by definition a necrotic material, but will partially remodel and incorporate into a new bony structure. This process involves a catabolic part with resorption of the dead bone graft by osteoclasts and an anabolic part with osteoblasts forming a new trabecular structure (van der Donk et al. 2002). Both the anabolic and catabolic processes can be manipulated by drugs (Little et al. 2007).

Bone morphogenetic proteins (BMPs) stimulate bone formation and bone allograft remodeling, and have been used to increase the remodeling of morselized and impacted bone graft both in animal models (McGee et al. 2004) and in human hip revisions (Kärrholm et al. 2006). However, BMPs also stimulate osteoclasts (Okamoto et al. 2006) and an increased resorption may cause a reduced load bearing capacity of the remodeling graft, resulting in mechanical failure (Tägil et al. 2003, Kärrholm et al. 2006). In contrast, bone graft that is not remodeled retains its load-bearing capacity (Parks and Engh 1997) but with time will be prone to fatigue fractures, which could weaken the implant fixation. We believe that remodeling of the bone graft is advantageous, but only if the bone can maintain its mechanical properties during the remodeling. Thus, when increased anabolism is desired in a mechanically loaded bone graft, a bisphosphonate might be used in addition to the bone anabolic BMP to control the resorption (Jeppsson et al. 2003).

Bisphosphonates have a strong affinity for calcium phosphate and are chemically bonded to the bone mineral (Rogers et al. 2000) regardless of whether they are administered systemically (Tägil et al. 2006) or locally (Aspenberg and Åstrand 2002). In consequence, a graft can be treated locally by simply soaking it in a bisphosphonate solution before implantation, thereby protecting the new-forming bone from the increased resorption caused by the BMP. We investigated whether it is possible to increase the formation of new bone in a bone graft by adding BMP-7 locally and at the same time protect the new-forming bone from resorption by local treatment with zoledronate, a potent third-generation bisphosphonate

## Methods

### The chamber

**Figure 1.**
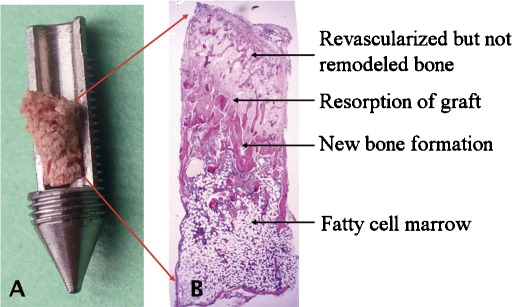
A. The bone conduction chamber with a freeze-dried graft. The graft is placed in the chamber and mesenchymal tissue grows in from the bottom upwards into the bone graft, which subsequently remodels. B. Histological picture (hematoxylin and eosin, ×20) of an untreated graft after 6 weeks in the chamber. Ingrowth occurs from the bottom of the picture as the graft is revascularized. Bone formation lags behind the vascular ingrowth and starts with an osteoclastic resorption of the bone graft, followed by a front of new forming bone. In this unloaded model, the new bone is almost entirely resorbed and finally remodeled into a fatty cell marrow.

We used a rat model with a cancellous allograft that remodels in vivo in a bone conduction chamber (BCC) (Aspenberg and Wang 1993) (Figure 1). The chamber consists of a threaded titanium cylinder, formed from 2 half-cylinders that are kept together by a hexagonal screw cap. The interior of the chamber is 7 mm long with a diameter of 2 mm. One end of the implant is screwed into the proximal tibia. At this end, there are 2 ingrowth openings each measuring 0.75 mm^2^, where tissue can grow in from the subcortical bone.

### Grafts

Pairs of structurally intact cancellous bone grafts were obtained from 34 female Sprague-Dawley rats (200 g; Taconic M&B A/S, Ry, Denmark). A cylindrical bone rod with a diameter of 2 mm was harvested in the axial direction from the knee joint with a hole cutter and the epiphysis was discarded. The rods were approximately 4–5 mm long. The grafts were kept sterile and freeze-dried for 24 h. At surgery, the grafts were thawed, placed in the chambers, and soaked with 8 μL of experimental solution or saline.

### Surgical procedure

34 male Sprague-Dawley rats (321–355 g; Taconic M&B) received bilateral chambers. The rats were anesthetized with diazepam and pentobarbitalnatrium. Antibiotic prophylaxis was given as 12.5 mg dihydrostreptomycin and 10 mg procaine benzylpenicillin. Under aseptic conditions, a longitudinal incision was made over the anteromedial aspect of the proximal tibial metaphysis. Holes were made with a drill and the chambers were screwed into position with the ingrowth holes situated subcortically. The wounds were closed, leaving the entire chamber subcutaneous. Postoperatively, analgesic was given as 4.5 μg buprenorphine subcutaneously. All animal handling was approved by the regional animal research ethics committee.

### Drug administration

BMP-7 (Osigraft; Stryker Biotech, Malmö, Sweden) was obtained as a powder with a concentration of 3.5 mg/1 g bovine collagen. The powder was diluted in saline to a concentration corresponding to 2 μg BMP-7 per graft. This amount corresponds to a clinical equivalent of 1 vial of BMP-7 to 1 morselized femoral head. Zoledronate (Zometa; Novartis, North Ryde, NSW, Australia) was obtained as a powder and was diluted in saline to a concentration corresponding to 4 μg zoledronate per graft. Immediately before insertion, each graft was soaked with 8 μL of either saline, BMP-7, zoledronate, or a combination of the latter two. To avoid a systemic effect of the bisphosphonate, the rats were randomized into 2 groups. One group received grafts treated with BMP-7 in the right leg and saline-treated in the left. In the other group, the rats received chambers containing both BMP-7 and zoledronate in the right leg and zoledronate alone in the left.

### Evaluation

The rats were killed after 6 weeks and the contents of the chambers were prepared for decalcified histology. The specimens were fixed in 4% formaldehyde, decalcified, dehydrated, and embedded in paraffin. They were cut parallel to the long axis of the chamber with a microtome and stained with hematoxylin and eosin. 3 sections from the middle of the specimens, each at 300 μm distance from the previous one, were used for histological and histomorphometric analyses. All slides in each experiment were investigated blind in random order.

**Figure 2.**
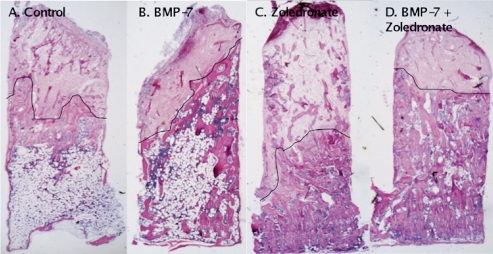
Histological specimens representing the 4 treatment groups after 6 weeks in the chamber (hematoxylin and eosin). Host tissue enters the graft through holes in the bottom of the chamber and ingrowth/remodeling occurs from the bottom upwards. The front of bone ingrowth is marked with a black line. A. Saline-treated chamber specimen with remodeled bone graft below the arrows. Most of the newly formed bone below the bone ingrowth front has already been remodeled into a fatty cell marrow. B. In the BMP-7-treated specimens, the bone ingrowth front has reached further into the graft than in the controls. C. In the zoledronate-treated specimens, the remodeled bone ***below*** the ingrowth front has a BV/TV (bone volume/total volume) value about 3 times higher than in the controls. D. Here, both BMP-7 and zoledronate were given and both the ingrowth distance and bone retention are increased compared to the control. The images have been chosen to be representative of the group median in terms of bone ingrowth and BV/TV value.

The area of the newly formed remodeled bone in each slide was measured using a digital system and a digitizer (Videoplan; Kontron Bildanalyse, Esching, Germany) by measuring the area at 20× screen magnification formed by the bottom and sides of the chamber and the distal bone ingrowth frontier. This new-formed remodeled bone area includes the new-formed marrow cavity within the graft as well as graft remnants that had been surrounded by new bone. The bone ingrowth frontier, the border between new-formed remodeled and non-remodeled bone, is clear (Figures 1 and 2). To compensate for a variable ingrowth distance of the bone ingrowth frontier into the graft, however, the mean bone ingrowth distance in each slide was calculated by dividing the area of the new-formed remodeled bone by the width of the specimen. The mean bone ingrowth distance for each animal was used as a final surrogate variable of bone anabolism, i.e. how far into the graft the remodeling took place.

The amount of remaining bone within the new-formed remodeled bone, behind the bone ingrowth frontier, was used as a surrogate variable of bone catabolism. A volume fraction estimation, equivalent to and expressed as BV/TV (bone volume/total volume), was made of both the remaining dead graft and living new-formed bone within this area by a point-count method ad modum Cavalieri, using a Merz grid ocular lens (Merz and Schenk 1970) with 6 × 6 crossing lines forming 36 points. Multiple sections of the new-formed remodeled bone of each graft were analyzed at 40× magnification using the point count method, from the bottom of the chamber to the bone ingrowth frontier. A distinction was made between dead graft bone and new living bone by evaluating matrix staining and the presence of osteocytes. The frequency of the crossings covering graft and newly formed bone tissue was recorded and expressed as a percentage of the total area measured (bone area/total area).

Arithmetically, the volume of the new-formed remodeled bone in the chamber can be expressed as a function of the mean bone ingrowth distance into the graft × π × r² where r is the radius of the chamber (1 mm). By multiplying BV/TV by the volume, we can calculate the total or relative amount of the retained new-formed and non-resorbed bone graft.

### Statistics

Results were analyzed non-parametrically, as normal distribution could not be assumed, using the Mann-Whitney U-test. We used SPSS software version 16.0 for Windows.

## Results

No wound infections occurred. 1 rat receiving zoledronate died during anesthesia. Tibial fractures occurred in 2 rats in the BMP-7 group during surgery and these were excluded. 1 chamber in the zoledronate group loosened and was excluded, thus leaving 17 grafts in the control group, 15 in the BMP-7 group, 15 in the zoledronate group, and 16 in the BMP-7 + zoledronate group.

### Histology

In all chambers, almost the entire graft had been revascularized at harvest. New bone had penetrated the graft and had formed a bone ingrowth frontier at varying distances into the graft (Figures 1 and 2). The frontier appeared to have advanced further into the grafts treated with BMP-7 (Figures 2b and 2d). Behind the frontier, the grafts were resorbed to varying extents and hematogenous or fat cell-rich bone marrow had formed. In the controls and BMP-7 group (Figures 2a and 2b), the grafts had been resorbed almost entirely. In contrast, the zoledronate-treated grafts appeared intact, with new bone formed around the graft trabeculae (Figures 2c and 2d). Both old graft bone and newly formed bone was retained in the zoledronate-treated samples. The difference in bone density was clearly visible. In all cases, vascularized fibrous tissue had penetrated further into the chamber ahead of the new bone.

### Histomorphometry

Both the group treated with zoledronate and the group treated with the combination of zoledronate and BMP-7 showed statistically significantly higher BV/TV values than the saline control and BMP-7 alone (Table 1). The relative proportion of old graft bone (dead bone/total bone) was 20% and 22% in the 2 zoledronate-treated groups, and 12% and 14% in the control group and the BMP-7-alone group. Bone ingrowth had reached further in the grafts treated with the combination of zoledronate and BMP-7, compared to zoledronate alone or saline (Table 2).

**Table 1.** The *catabolic* part of the bone remodeling, illustrated by the BV/TV value (bone volume/total volume; expressed as median (range)). BV/TV is mainly influenced by the addition of a bisphosphonate, which stalls catabolism. Comparisons are shown unpaired between the right sides, treated with BMP-7 and the left sides, treated with saline, for both the zoledronate (ZA) group and the control group. The amount of new-formed bone on the old graft is given for comparison

	Saline (n = 17)	ZA (n = 15)	p-value	BMP-7 (n = 15)	BMP-7 + ZA (n = 16)	p-value
BV/TV, %	16 (10–40)	56 (37–68)	< 0.001	14 (2–54)	50 (40–57) **^a^**	< 0.001
New-formed	14 (7–34)	47 (23–58)		11 (2–43)	39 (26–56)	
Retained graft	2 (0–12)	11 (5–17)		2 (0–10)	11 (7–18)	

**^a^** p < 0.001 vs. control.

**Table 2.** The *anabolic* part of the bone remodeling, illustrated by the bone ingrowth distance of new bone into the chamber (median (range)). Bone ingrowth distance is mainly influenced by the addition of a bone proliferation and differentiation inducer such as BMP-7. Comparisons have been made pairwise (15 complete pairs) between the right side, treated with BMP-7, and the left side, treated with saline, for both the zoledronate (ZA) group and the control group

	Saline	BMP-7	p-value	ZA	ZA + BMP-7	p-value
Ingrowth, mm	2.6 (1.5–4.4)	3.0 (1.9–4.4)	0.07	2.3 (1.0–3.5)	3.5 (2.4–5.8) **^a^**	< 0.003

**^a^** p = 0.002 vs. control.

In total, the net amount of remodeled and retained bone in the grafts increased to 5.5 mm³ when BMP-7 and zoledronate were combined, as compared to 1.3 mm³ in the control, a more than fourfold increase. In the zoledronate-alone group the total retained bone volume was 4.0 mm³, and in the BMP-7-alone group it was 1.3 mm³.

## Discussion

In hip revisions using morselized and impacted allograft, the remodeling is slow and incomplete (van der Donk et al. 2002, Linder 2000). A mixed pattern, with areas of remodeled bone, revascularized bone without remodeling, and inert, non-revascularized and non-remodeled bone is found histologically (Linder 2000). These histological findings are similar to the various areas within the bone chamber (Figures 1 and 2). To improve graft remodeling, drugs have been used to speed up the process and quicker form a living support to the implant and cement construct (McGee et al. 2004, Kärrholm et al. 2006). BMP-7 has been shown to stimulate graft remodeling in several experimental studies but—something that is perhaps less well known—it also appears to activate osteoclasts, leading to an increased amount of resorption (Okamoto et al. 2006).

**Figure 3.**
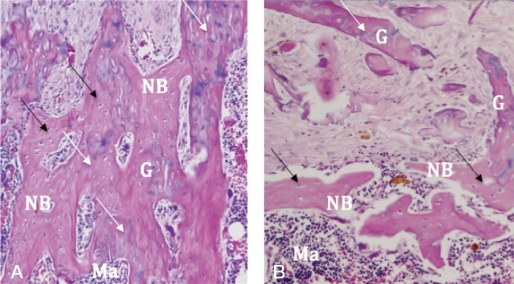
Bisphosphonate-treated specimen to the left (A) and saline-treated control to the right (B) (hematoxylin and eosin, ×40). A. Note how the new-formed bone (NB), identifiable by the existing cell nuclei (black arrows), covers the old graft bone (G) with its lost nuclei (white arrows). Due to the delayed resorption, an old bone–new bone construct is formed. B. In the saline-treated control, such a construct does not form as extensively. The graft bone (G) is resorbed and replaced by new bone (NB), which is in turn immediately resorbed, being replaced by a hematogenous/fat cell marrow (Ma).

In the present rat bone chamber experiment, BMP-7 sped up the ingrowth and the remodeling front advanced deeper into the graft than it would have without BMP-7. On the other hand, all the bone that formed was more or less totally resorbed behind the thin new-forming bone front. Since bone strength correlates mainly with BV/TV ratio (Carter and Spengler 1978); a full remodeling, although achieved earlier due to the BMP, may be less advantageous regarding load bearing (Burchardt 1983). By adding a bisphosphonate, this unwanted resorption was controlled, both in the BMP-treated and saline-treated grafts. The BV/TV ratio of the remodeled bone increased and did so without disturbing the new bone formation, as seen and feared for in other studies (Jeppsson et al. 2003, Jakobsen et al. 2007, Baas et al. 2008). In the bisphosphonate-treated specimens, sometimes the graft bone was not resorbed but was instead covered with the new-forming bone, thus forming an integrated construct of old and new bone (Figure 3). The combination of an anabolic drug (BMP-7) and an anti-catabolic drug (zoledronate) resulted in both an increased ingrowth distance of new bone into the bone graft and a dramatically increased BV/TV value. In total, more than 4 times more bone was present after remodeling in the chamber when the combination of BMP-7 and zoledronate was used compared to saline-treated control graft.

Several advantages can be obtained by combining a locally applied BMP with a locally applied bisphosphonate, instead of systemic administration. With local administration, the drug is added to the graft already during the operation. All of the graft will be treated independently of the level of revascularization, and all bone binding sites will be saturated with chemically bound zoledronate, thus protecting the graft from resorption until completely remodeled. With local administration, poor patient compliance is avoided (Rabenda et al. 2008). Furthermore, the systemic effects of an intravenous bisphosphonate can be avoided and the total dose used kept smaller than with systemic treatment.

The bone chamber is a simple model to understand basic bone remodeling. Since the remodeling occurs from the bottom of the chamber and upwards in a certain sequence, it is easy to identify the different and sequential steps of graft remodeling. The chamber does, however, have limitations and the results might not be generalizable for clinical use. With other animal models, concerns have been raised regarding local treatment (Jakobsen et al. 2007), implying risks of an anabolic deficiency caused by the bisphosphonate also when combined with BMP. In vitro studies have demonstrated that bisphosphonates can inhibit osteoblastic cell metabolism (Iwata et al. 2006) and induce apoptosis in a wide range of cells, including osteoblasts. In vivo, bisphosphonates in clinically used doses become chemically bound to bone and are metabolized almost exclusively by osteoclasts. However, with excessive amounts of unbound bisphosphonate released into the tissue, an anti-anabolic effect might occur (Schindeler and Little 2007). In a canine implant study, 2.5-mm peri-implant gaps were filled with impacted bone graft with or without bisphosphonate. A reduced degree of mechanical fixation was found in the bisphosphonate group due to decreased bone formation and delayed removal of debris (Jakobsen et al. 2007). In that study, just as in ours, the graft was not rinsed from excessive bisphosphonate fluid before impaction and the authors speculated that the reason for the inferior mechanical strength might be due to an unwanted anti-anabolic effect on top of the desired anti-catabolic effect. In another study, investigating BMP-2 and pamidronate in an impaction grafting model, not only the substances alone but also the combination was inferior to the saline control as pamidronate virtually blocked bone metabolism, even when combined with BMP-2 (Baas et al. 2008).

In most previous studies from our laboratory, any excess of bisphosphonate has been washed away through rinsing of the graft. In the present study, this was not in the protocol because rinsing would have washed away the non-binding BMP-7, which was also added locally. Thus, high concentrations of zoledronate might have occurred locally, having a negative effect on bone formation and bone ingrowth. In the zoledronate group, the ingrowth distance appeared somewhat shorter (but not statistically significantly so) compared to the control group, indicating such a negative effect. The ingrowth distance was indeed shorter than all previous pooled chamber studies done in our laboratory using systemic bisphosphonates. Perhaps local bisphosphonate treatment would impair bone formation to a greater extent than systemic, a hypothesis that deserves further investigation.

Administration of zoledronate locally increases bone graft density after remodeling. It has a low risk of systemic side effects but an equivalent effect on resorption compared to previous studies using systemic zoledronate (Harding et al. 2008). There is a risk of reduced bone formation with locally administered bisphosphonates, which in our model could easily be compensated for by local administration of BMP at the same time. However, further investigations must be performed before local treatment can be tried clinically, especially analysis of whether the mechanical strength is increased by the increased bone formation and density.
